# Combination of tumor markers predicts progression and pathological response in patients with locally advanced gastric cancer after neoadjuvant chemotherapy treatment

**DOI:** 10.1186/s12876-021-01785-7

**Published:** 2021-07-10

**Authors:** Zining Liu, Yinkui Wang, Fei Shan, Xiangji Ying, Yan Zhang, Shuangxi Li, Yongning Jia, Rulin Miao, Kan Xue, Zhemin Li, Ziyu Li, Jiafu Ji

**Affiliations:** Key Laboratory of Carcinogenesis and Translational Research (Ministry of Education/Beijing), Gastrointestinal Cancer Center, Peking University Cancer Hospital and Institute, Beijing, 100142 China

**Keywords:** Gastric cancer, Neoadjuvant chemotherapy, Tumor marker, Time-dependent ROC, Survival

## Abstract

**Background:**

The prognostic values of preoperative tumor markers (TMs) remain elusive in patients with locally advanced gastric cancer (LAGC) after neoadjuvant chemotherapy treatment (NACT). This study aimed to assess and establish a novel scoring system incorporating carcinoembryonic antigen (CEA), carbohydrate antigen 19-9 (CA19-9), carbohydrate antigen 72-4 (CA72-4) to enhance prognostic accuracy for progression-free survival (PFS) and pathological response (pCR).

**Methods:**

Patients' data were retrospectively analyzed from December 2006 to December 2017 in our center. The cutoff value of TMs was determined using the time-dependent receiver operating test characteristics method. These three TMs were allocated 1 point each for the post neoadjuvant chemotherapy combination of tumor markers (post-NACT CTM) scores. The training group comprised 533 patients, responsible for full analysis, and the validation group comprised 137 patients based on the selection protocol.

**Results:**

Of 533 enrolled patients, 138, 233, 117, and 45 patients scored 0, 1, 2, 3 respectively. The 3-year PFS rate Multivariate analysis revealed that post-NACT CTM score was an independent predictor of PFS (0 vs. 1, HR: 1.34, 95% CI: 0.92–1.96, *P* = 0.128; 0 vs. 2, HR: 2.03, 95% CI: 1.35–3.05, *P* = 0.001; 0 vs. 3, HR: 2.98, 95% CI: 1.83–4.86, *P* < 0.001). The time-dependent area under curve (AUC) revealed a consistent highest level for post-NACT CTM than other three single TMs. Lower post-NACT CTM score significantly correlated with higher pCR rate based on multivariate logistic regression (2/3 vs. 1, OR: 2.77, 95% CI: 0.90–8.53, *P* = 0.077; 2/3 vs. 0, OR: 4.33, 95% CI: 1.38–13.61, *P* = 0.012). A nomogram was formed with both internal and external validation.

**Conclusions:**

The post-NACT CTM score system served as a strong independent predictor for PFS and pCR in LAGC patients who received NACT. Further population-based studies are required to confirm our results.

**Supplementary Information:**

The online version contains supplementary material available at 10.1186/s12876-021-01785-7.

## Introduction

Gastric cancer (GC) is the fifth most commonly diagnosed neoplasm, accounting for 5.7% of all cancers worldwide [1]. In China, GC is often diagnosed at advanced stage and has poor prognosis. Although surgery is the only curative approach for patients with locally advanced gastric cancer (LAGC), multimodal therapy has shown to be superior to surgery alone. Curative treatment LAGC usually entails neoadjuvant chemotherapy (NACT) followed by surgery and postoperative adjuvant chemotherapy [2].

Despite the introduced 8th AJCC post-neoadjuvant therapy stage (ypTNM) is effective in predicting long-term survival, the prognosis of GC may be affected by other individual factors like tumor differentiation, behavior and genetic abnormalities, etc. [3, 4].

Throughout these years, studies have revealed that levels of tumor markers (TMs) may reflect tumor burden in certain circumstances and can be used in staging, prognostication or prediction of response to therapy [5–7]. Based on the NACT modality, the benchmarked TMs levels were usually based on the pretreating settings, while most studies focused on the alteration of TMs level during preoperative treatment. The sad truth is, nearly half of patients are pathological poor-responders, changed values of TMs during NACT are often minimal [8, 9]. Moreover, the measurement of response for NACT is more likely to depend on pathological evaluations instead of changed TMs levels [10]. In this process, the clinical value of TMs in measuring residual tumor burden after NACT is neglected. As the prognostic values for preoperative TMs have been vastly investigated in previous studies, we have reasons to suppose that the post-NACT TMs values may have similar predictive strength in patients' survival as immediate feedback for residual tumor load [11].

However, a single TM value always has low rate of sensitivity and specificity and can be easily affected by noncancerous conditions [12]. To achieve a higher prognostic value and put TMs into applications, a combined diagnosis of TMs is to be at hand. The most commonly used tumor markers of gastric cancer were CEA, CA19-9, CA72.4, and CA125, sometimes plus AFP and CA242. Liu et al. once found the potential numeric association between the combination of tumor markers (CTM) and the intrinsic tumor parameters that could evaluate the probability of overall survival (OS) in patients with direct curative surgery [7]. The method was revealed to be effective with high feasibility, which can be potentially applied to patients undergoing NACT measuring the tumor load and predicting prognosis.

Given the considerations above, whether a benchmark value for TMs can classify the prognosis for LAGC patients with NACT is still in the mist. Hereby, we introduce a post-NACT combination of serum tumor markers (post-NACT CTM) involving CEA, CA 19-9, and CA 72-4. Other TMs were counted out following the result from a preliminary dataset (data not shown). We aim to see the clinical value in using absolute posttreatment levels of TMs to predict patients' risk of progression (primary outcome) and the rate of pathological response (secondary outcome). A nomogram was also plotted based on the independent predictors.

## Methods

### Patients

We obtained data from a prospective database of all patients who started NACT at the Peking University Cancer Hospital and Institute from December 1, 2006, to December 1, 2017. The determination of clinical stages, design for treatment route, preoperative assessment, and prompt intervention for adverse events were managed by the multidisciplinary team (MDT).

Study inclusion criteria included: (1) proven diagnosis of gastric adenocarcinoma by preoperative biopsy; (2) complete medical record and document with TMs record before NACT initiation; (3) no signs of distant metastasis at first visit; (4) curative gastrectomy was performed.

**Fig. 1:**
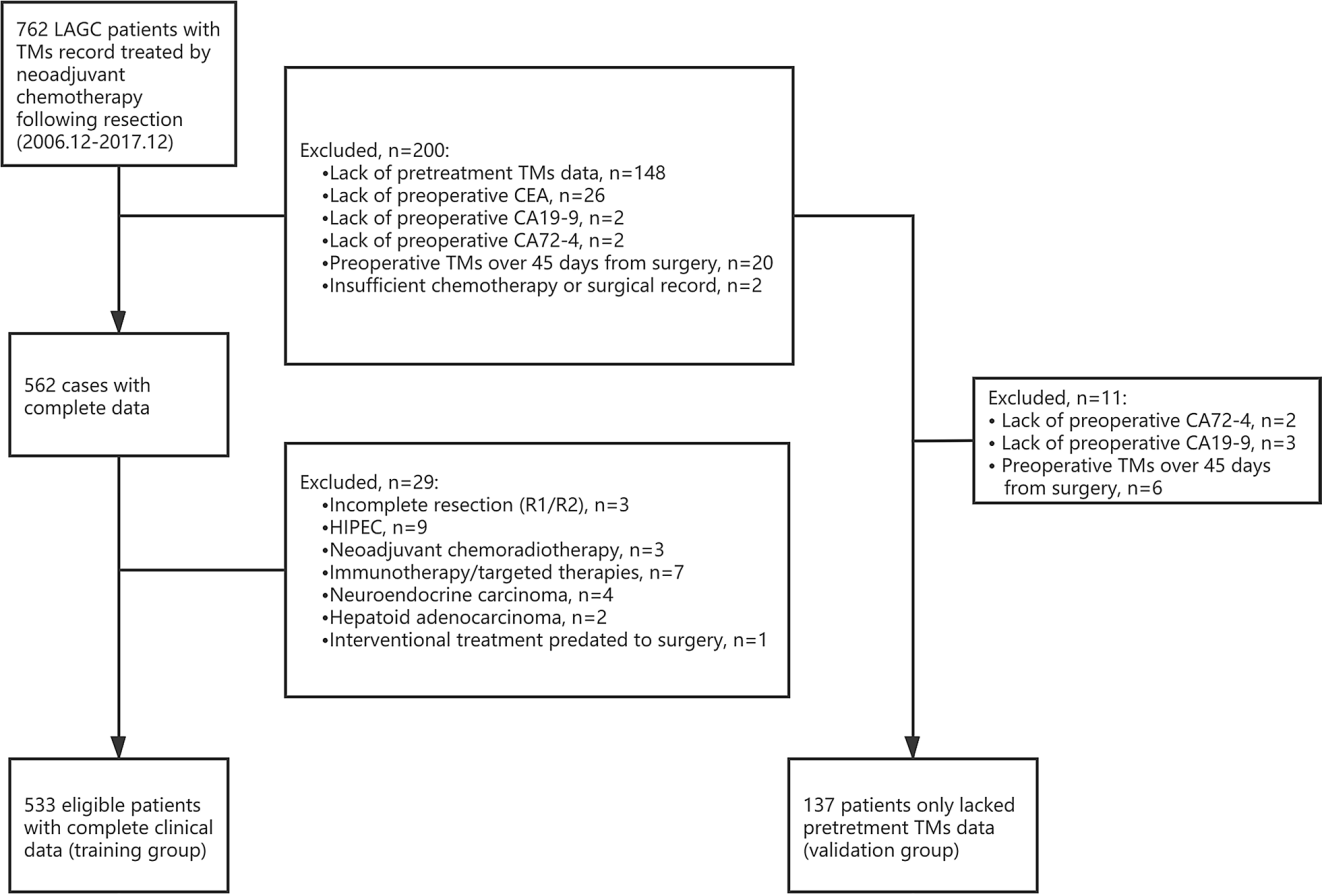
Selection of patients for inclusion

The exclusion criteria for the training dataset were as follows: (1) Incomplete post-NACT tumor marker record including CEA, CA19-9 and CA72-4; (2) patients who received preoperative radiotherapy, targeted therapy and interventional chemotherapy; (3) patients who received intraperitoneal chemotherapy or hyperthermia intraperitoneal chemotherapy before curative resection; (4) patients with R1/R2 resection; (5) patients with D0/D1/D1 + lymphadenectomy; (6) Prior history of gastrointestinal tumors; (7) Inconsistent of non-adenomas diagnosis confirmed by postoperative pathology. The availability of pre-NACT TMs data separated patients into a training group and validation group. Finally, 533 patients were eligible for main analysis, while 146 cases without pre-NACT TMs records served as the validation samples for the nomogram model (Fig. 1).

### Regimen and radical surgery

All patients received at least two cycles of chemotherapy, in the form of 5-Fu based combined regimens before surgery's radicalness. The majority of patients were treated with 5-Fu-based plus oxaliplatin doublets: SOX (oxaliplatin plus S-1) in 254 patients, CapeOX (oxaliplatin plus capecitabine) in 123 patients, and FOLFOX (oxaliplatin plus 5-Fu/4-Lv) in 105 patients. Some received 5-Fu-based plus paclitaxel doublets: PX (paclitaxel plus capecitabine) in 18 patients, PS (paclitaxel plus S-1) in 15 patients. The rest of the other regimens involving 18 patients were as follows: POS (paclitaxel, oxaliplatin, and S-1) in 10, CS (cisplatin plus S-1) in four, IRIS (irinotecan plus S-1) in two, EOX (epirubicin, oxaliplatin and capecitabine) in one, DCF (paclitaxel, cisplatin and 5-Fu) in one, respectively. Additional file 1: Table S1 described detailed dosing regimens. To assess the influence of the treatment duration, the three 14-day cycles of FOLFOX or POS were calculated as two 21-day cycles and were transformed based on the rounding strategies, consistent with the other 5-Fu-based regimens. Dosage reduction or withdrawal was applied in cases of severe adverse events during chemotherapy, as determined by clinicians. After two to three chemotherapy cycles, the antitumor effect was evaluated using abdominal computed tomography (CT). Basically, two or three alignment cycles were performed. The therapy was prematurely terminated in cases of disease progression. Otherwise, gastrectomy or continued NACT was considered after obtaining informed consent and approval from patients. Subtotal or total gastrectomy plus D2 lymphadenectomy was performed according to the Japanese Gastric Cancer Association (JGCA) guideline [13].

### Data collection

The patient characteristics, including age, body mass index (BMI), gender, American Society of Anesthesiologists score (ASA), ECOG performance status, comorbidities, tumor location, tumor diameter (on short axis), differentiation grade, vascular involvement, posttherapy pathological (yp) TNM stage according to the 8th American Joint Committee on Cancer (AJCC) guideline, type of resection, complications graded by Clavien-Dindo classification system, total cycles of chemotherapy, date of NACT initiation, date of surgery, date of adjuvant chemotherapy initiation, date of progression [14, 15]. The value of three TMs in each patient was obtained at the time of the first visit and within 45 days before surgery.

### Histopathology analysis

All pathological examinations were undertaken by two experienced gastrointestinal pathologists, who were blinded to the group assignment. We assessed efficacy by using the pathological complete response (pCR) rate according to National Comprehensive Cancer Network (NCCN) guidelines [16]. pCR was defined as the elimination of any viable residual tumor cell in the resected primary tumor and adjacent lymph nodes (ypT0N0).

### Follow-up

Patients were followed up regularly via physical examination, radiological examination, endoscopic examination, and laboratory examination or telephone call when faced with the inconvenience. These examinations were performed quarterly during the first 2 years, then every 6 months until the fifth year, and then once a year.

### Tumor markers and scoring methods

The level of CEA, CA19‐9, and CA72-4 was obtained via laboratory analysis of the patients' routine blood test at initial diagnosis with the upper normal values 5 ng/mL, 35 ng/mL and 6.9 ng/L, respectively. The optimal cutoff values for CEA, CA19-9 and CA72-4 were determined by the highest Youden index using time-dependent receiver operating curve (ROC) analysis with "survivalROC" package based on 3-year PFS with Kaplan–Meier method [17]. The output of 95% confidence interval and compared of AUC were based on the inverse probability of censoring weighting (IPCW) approach implemented in the package "timeROC" developed by Blanche et al. [18]. Because the analysis indicated that a TM level exceeding the cutoff value was associated with shorter PFS, each tumor marker was allocated 1 point of post-NACT CTM score.

### Statistical analysis

Continuous variables were summarized as median (IQR) and were compared across groups using the Wilcoxon-rank-sum or Kruskal–Wallis test for two or more group comparisons for continuous variables. Categorical variables were analyzed using the Chi-squared test or Fisher's exact test. The areas under the ROC curve (AUCs) of the TMs for predicting 1-year, 3-year, and 5-year PFS were calculated and used for comparisons with other models, which has been described above. The relationships between clinical and pathological factors and long-term PFS were assessed using log-rank tests and Cox proportional hazard model. Tumor or treatment characteristics that achieved a *P* value < 0.10 in univariate analysis were included in the multivariate analysis. To examine parameters with high collinearity, we used pairwise relationship correlation coefficients (no coefficient greater than |0.4|) to assess collinearity among predictors. Based on the univariate and multivariate Cox regression analyses, we established nomograms predicting 1-, 3- and 5-year PFS. For the convenience of clinical application, the model was represented as a nomogram using R software's "rms" package [19]. The predictive accuracy of the model was internally validated with Harrell's C-statistic (C-index) using 1000 bootstrap repetitions ranging from which 0.5 (perfect discordance) to 1 (ideal concordance). A calibration plot was generated to examine the performance characteristics of nomograms. Logistic regression was used to evaluate time-independent outcomes. Testing for trends can be applied based on various statistical hypothesis when necessary. We conduct a Spearman correlation analysis to assess the relationship between factors identified in the risk assessment and post-NACT scores. Conventionally, we interpreted a correlation coefficient of < 0.3 as weak, 0.3–0.7 as moderate and > 0.7 as strong. For all analyses, *P* < 0.05 was considered statistically significant. Statistical analyses were performed using SE STATA (Stata Statistical Software, release 15.1; Stata Corp, College Station, TX, USA) or R (R version 3.6.2).

## Results

### Patients characteristics and establishment of the scoring system

The clinicopathological characteristics of the patients and their predicted 1-year and 3-year PFS KM rate with 95% CI based on each character are summarized in Table 1. The median follow-up time was 63 (IQR 38–87) months. Median post-NACT CEA, CA19-9 and CA72-4 were 2.42 (IQR: 1.63–4.21), 12.88 (IQR: 7.24–24.64) and 2.92 (IQR: 1.54–7.12), respectively.

**Table 1:** Demographic and clinicopathologic characteristics of the study population with 1-year and 3-year PFS

Characteristics	N (%)	1-year PFS (95% CI)	*P*	3-year PFS (95% CI)	*P*
Age	60 (53–66)		0.610		0.571
≤ 60	282 (52.91)	84.75 (80.00–88.46)		63.66 (57.69–69.03)	
> 60	251 (47.09)	86.45 (81.57–90.12)		61.15 (54.68–66.98)	
BMI	23.44 (21.30–25.43)		0.084		0.007
≤ 23.9	305 (57.22)	83.28 (78.60–87.02)		57.21 (51.32–62.66)	
> 23.9	228 (42.78)	88.60 (83.71–92.09)		69.38 (62.89–74.96)	
Sex			0.697		0.322
Male	413 (77.49)	85.23 (81.43–88.31)		63.56 (58.62–68.08)	
Female	120 (22.51)	86.67 (79.16–91.61)		58.63 (49.20–66.91)	
ASA score			0.220		0.685
1	49 (9.19)	81.63 (67.67–89.99)		68.98 (53.86–80.01)	
2	418 (78.42)	84.93 (81.13–88.02)		62.64 (57.73–67.15)	
3	66 (12.38)	92.42 (82.75–96.77)		57.31 (44.46–68.22)	
ECOG			0.063		< 0.001
0	384 (72.05)	87.24 (83.47–90.20)		68.96 (64.00–73.39)	
≥ 1	149 (27.95)	81.21 (73.96–86.62)		46.02 (37.74–53.89)	
Comorbidities			0.577		0.420
0	354 (66.42)	86.16 (82.10–89.35)		63.73 (58.40–68.56)	
≥ 1	179 (33.58)	84.36 (78.15–88.92)		60.02 (52.31–66.88)	
Location			0.002		< 0.001
Upper	165 (30.96)	87.27 (81.15–91.51)		64.37 (56.47–71.21)	
Middle	77 (14.45)	88.31 (78.74–93.74)		62.87 (50.85–72.73)	
Lower	266 (49.91)	86.09 (81.32–89.72)		65.34 (59.17–70.82)	
Diffuse	25 (4.69)	60.00 (38.45–76.11)		18.00 (5.82–35.57)	
Diameter (cm)	2 (1.5–3.5)		< 0.001		< 0.001
≤ 2	289 (54.22)	90.31 (86.28–93.21)		75.38 (69.87–80.03)	
2–5	190 (35.65)	84.74 (78.79–89.13)		52.28 (44.81–59.21)	
≥ 5	54 (10.13)	62.96 (48.68–74.28)		29.38 (17.90–41.81)	
Differentiation			0.044		0.080
Well/Moderate	163 (30.58)	90.18 (84.48–93.87)		67.68 (59.71–74.42)	
Poor	370 (69.42)	83.51 (79.32–86.92)		60.20 (54.95–65.04)	
ypT			0.004		< 0.001
T0	39 (7.32)	97.44 (83.16–99.63)		92.08 (77.39–97.38)	
T1	54 (10.13)	92.59 (81.46–97.15)		81.63 (67.36–90.09)	
T2	80 (15.01)	91.25 (82.52–95.73)		79.70 (68.99–87.05)	
T3	119 (22.33)	86.55 (78.99–91.54)		62.60 (52.98–70.80)	
T4	241 (45.22)	79.67 (74.01–84.22)		47.82 (41.35–53.99)	
ypN			< 0.001		< 0.001
N0	233 (43.71)	95.71 (92.17–97.67)		83.25 (77.70–87.53)	
N1	109 (20.45)	88.99 (81.43–93.59)		68.96 (59.17–76.86)	
N2	86 (16.14)	81.40 (71.44–88.16)		50.59 (39.49–60.67)	
N3	105 (19.70)	62.86 (52.87–71.30)		20.80 (13.62–29.03)	
Resection type			< 0.001		< 0.001
Subtotal	309 (57.97)	89.97 (86.04–92.84)		68.54 (62.95–73.48)	
Total	224 (42.03)	79.46 (73.56–84.19)		54.14 (47.29–60.49)	
Adjuvant chemotherapy			0.007		< 0.001
No	96 (18.01)	77.08 (67.32–84.27)		46.68 (36.24–56.45)	
Yes	437 (81.99)	87.41 (83.93–90.19)		65.96 (61.24–70.25)	
Cycle of NACT			0.233		0.110
≤ 3	478 (89.68)	84.94 (81.41–87.85)		61.21 (56.61–65.47)	
> 3	55 (10.32)	90.91 (79.53–96.11)		74.45 (60.70–84.00)	
Clavien–Dindo			0.077		0.100
0–II	455 (85.37)	86.59 (83.11–89.41)		63.59 (58.90–67.90)	
III–IV	78 (14.63)	79.49 (68.72–86.89)		56.05 (44.26–66.28)	
Post-NACT CEA	2.42 (1.63–4.21)		< 0.001		< 0.001
≤ 5.72	447 (83.86)	88.14 (84.77–90.81)		67.48 (62.84–71.67)	
> 5.72	86 (16.14)	72.09 (61.32–80.34)		36.59 (26.43–46.78)	
Post-NACT CA19-9	12.88 (7.24–24.64)		< 0.001		< 0.001
≤ 15.00	311 (58.35)	90.35 (86.49–93.15)		70.88 (65.42–75.65)	
> 15.00	222 (41.56)	78.83 (72.85–83.64)		50.58 (43.67–57.08)	
Post-NACT CA72-4	2.92 (1.54–7.12)		< 0.001		< 0.001
≤ 2.60	244 (44.78)	92.62 (88.55–95.29)		73.29 (67.18–78.44)	
> 2.60	289 (54.22)	79.58 (74.46–83.79)		53.31 (47.27–58.98)	
Post-NACT CTM			< 0.001		< 0.001
0	140 (26.27)	95.71 (90.71–98.05)		81.35 (73.82–86.90)	
1	233 (43.71)	88.84 (84.05–92.26)		65.22 (58.53–71.11)	
2	116 (21.76)	73.28 (64.23–80.38)		46.77 (37.38–55.60)	
3	44 (8.26)	68.18 (52.27–79.76)		27.70 (15.13–41.78)	

Values in parentheses are percentages unless indicated otherwise; ASA, American Society of Anesthesiologists; CI, confidence interval; BMI, Body Mass Index; CA19-9, carbohydrate antigen 19-9; CA72-4, carbohydrate antigen 72-4; CEA, carcinoembryonic antigen; CI, confidence interval; CTM, combination of tumor markers; ASA, American Society of Anesthesiologists; ECOG, Eastern Cooperative Oncology Group; NACT, neoadjuvant chemotherapy; PFS, progression-free survival; NACT, neoadjuvant chemotherapy; CEA, carcinoembryonic antigen; CA19-9, carbohydrate antigen 19-9; CA72-4, carbohydrate antigen 72-4; *P* value for log-rank test

The time-dependent ROC for the 3-year PFS was used to define the optimal cutoff of CEA, CA19-9, and CA72-4, with the AUC 0.592 (cutoff value: 5.72 ng/ml), 0. 620 (cutoff value: 15.00 ng/ml), and 0.597 (cutoff value: 2.60 ng/ml), respectively (Table 2). Thus, the combination of optimal cutoff values for the three tumor markers in identifying patients' prognosis was introduced as post-NACT CTM scores based on previous descriptions. Of the 533 patients, 140 (26.27%) patients scored 0, whilst 233 (43.71%), 116 (21.76%), and 44 (8.26%) patients scored 1, 2 and 3 respectively. Time-dependent ROC for 1-year, 3-year and 5-year PFS are presented in Additional file 2: Fig. S1.

**Table 2:** Performance of candidate TMs and post-NACT CTM for predicting risk of 3-year PFS

	AUC (95% CI)	*P*	*P* (compare to CTM)
CEA	0.592 (0.552–0.658)	0.001	0.003
CA19-9	0.620 (0.569–0.673)	< 0.001	0.011
CA72-4	0.597 (0.542–0.646)	< 0.001	< 0.001
Post-NACT CTM	0.681 (0.635–0.727)	< 0.001	1.000

CI, confidence interval; AUC, area under curve; CEA, carcinoembryonic antigen; CA19-9, carbohydrate antigen 19-9; CA72-4, carbohydrate antigen 72-4; CEA, carcinoembryonic antigen; CI, confidence interval; CTM, combination of tumor markers; Calculation of confidence interval and *P* value are based on Inverse Probability of Censoring Weighting method

**Fig. 2:**
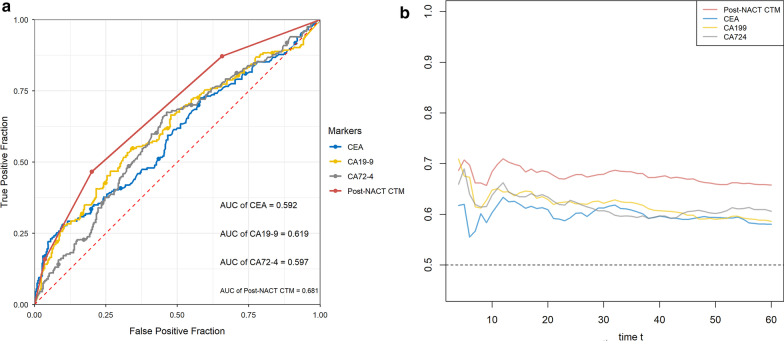
**a** Time-dependent ROC curves for the CEA, CA19-9, CA72-4 as well as the post-NACT CTM according to 3-year progression-free survival (PFS). The AUCs for the four indicators is presented in the plot. While all performed statistical significance compared to reference, Compared with the AUC in the CEA, CA19-9 and CA72-4 group, the AUC in combination group was significantly increased (vs. CEA, *P* = 0.003; vs. CA19-9, *P* = 0.011; vs. CA72-4 *P* < 0.001). **b** Accuracy of recurrence prediction as a function of follow-up months. The time-dependent AUC values for three TMs and post-NACT CTM were computed for each time point separated by month. The prediction accuracy was largely stable in the combination method except during the first three after surgery

We compare the AUC of the three tumor markers and the post-NACT CTM at 3-year PFS (Fig. 2a). The AUC of the combined diagnostic method was 0.677, significantly different from the AUC of the individual diagnostic method (*P* = 0.003, *P* = 0.011 and *P* < 0.001, respectively, Table 2). On the other hand, the AUC values were comparable for the addition of three tumor markers (CEA vs. CA19-9, *P* = 0.616; CA19-9 vs. CA72-4, *P* = 0.432; CEA vs. CA72-4, *P* = 0.740). Figure 2b plots the AUC for time-dependent ROC performance within 5 years at continuous-time points for the four measurements. It can be seen from the curve that the time-dependent AUC of post-NACT CTM keeps the highest for progression across all the time points while the AUC curve of CEA, CA19-9 and CA72-4 are in similar positions and get crossed over time, indicating that post-NACT CTM can better predict progression at a random exit time.

### Performance of the post-NACT CTM for predicting the risk of progression

**Fig. 3:**
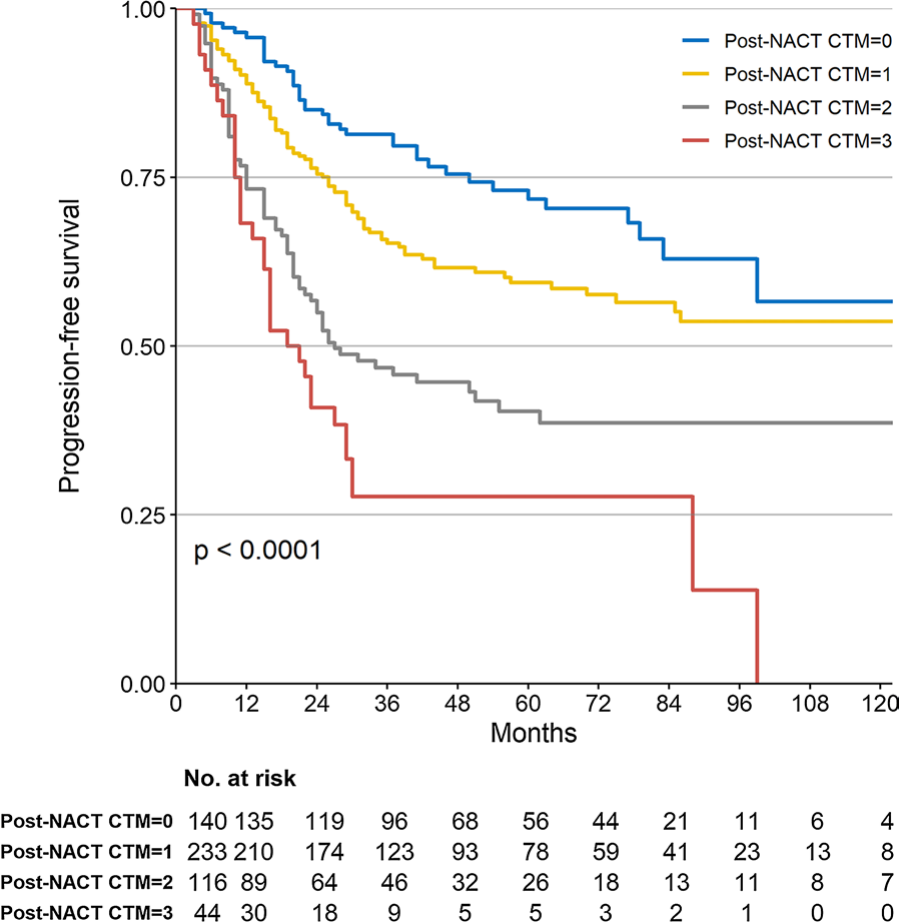
Kaplan–Meier estimates for Progression-free survival by post-NACT CTM group (log-rank test of equality, *P* < 0.001). The numbers below the graph indicate the number of subjects at each follow-up

At the time of the analysis, 232 had experienced a recurrence. Kaplan–Meier curves for PFS and PFS stratified by post-NACT CTM are presented in Fig. 3. There is significant stratification between groups in PFS (log-rank *P* < 0.001, P_trend_ < 0.001).

To further examine the prognostic values of post-NACT CTM with regard to the survival in all patients, the multivariate Cox proportional hazards model was formulated, adjusting for potential confounders based on *P* < 0.10 in BMI, ECOG, tumor location, diameter, differentiation, LVI, ypTNM stages, resection types, complications, and post-NACT CTM score in univariate analyses (Table 3). At multivariate analysis, LVI and type of resection were excluded from the variables because of their association with the ypN (r = 0.546, *P* < 0.001) and tumor location (r = − 0.402, *P* < 0.001), respectively. Of note, because of their association (r = 0.445, *P* < 0.001), ypT and ypN were integrated as ypTNM stage for further analysis. Results from the multivariate analysis indicated that post-NACT CTM score was independent predictor of PFS (0 vs. 1, HR: 1.37, 95% CI: 0.94–2.00, *P* = 0.099; 0 vs. 2, HR: 2.07, 95% CI: 1.37–3.10, *P* < 0.001; 0 vs. 3, HR: 3.11, 95% CI: 1.91–5.07, *P* < 0.001) along with ECOG, tumor location, diameter, ypTNM stage and adjuvant chemotherapy. The prognostic significance of post-NACT CTM was still performed well in patients with ypTNM stage either in 0/I (0 vs. 1, HR: 2.60, 95% CI: 0.69–9.73, *P* = 0.156; 0 vs. 2, HR: 4.84, 95% CI: 0.85–27.51, *P* = 0.075; 0 vs. 3, HR: 17.96, 95% CI: 2.06–156.58, *P* = 0.009) or in stage II/III (0 vs. 1, HR: 1.18, 95% CI: 0.80–1.75, *P* = 0.408; 0 vs. 2, HR: 2.07, 95% CI: 1.36–3.15, *P* = 0.001; 0 vs. 3, HR: 2.88, 95% CI: 1.74–4.76, *P* < 0.001).

**Table 3:** Univariate and multivariate analyses for progression-free survival using a Cox proportional hazards model

Variables	PFS			
	Univariate		Multivariate	
	Hazard ratio*	*P*	Hazard ratio*	*P*
Age (years)				
≤ 60	1.00			
> 60	1.07 (0.83–1.38)	0.613		
BMI (kg/m^2^)				
≤ 23.9	1.00		1.00	
> 23.9	0.72 (0.55–0.94)	0.015	0.83 (0.63–1.09)	0.184
Gender				
Male	1.00			
Female	1.02 (0.75–1.39)	0.903		
ASA score				
1	1.00			
2	1.23 (0.76–2.00)	0.402		
3	1.39 (0.79–2.46)	0.255		
ECOG (per 1 increase)	1.61 (1.32–1.96)	< 0.001	1.27 (1.01–1.60)	0.043
Comorbidities				
No	1.00			
Yes	1.13 (0.87–1.48)	0.363		
Location				
Localized	1.00	0.383		
Diffused	3.81 (2.44–5.94)	< 0.001		
Location				
Upper	1.00		1.00	
Middle	1.10 (0.73–1.66)	0.633	1.44 (0.93–2.23)	0.100
Lower	0.94 (0.70–1.27)	0.686	1.25 (0.90–1.72)	0.181
Diffused	3.74 (2.31–6.08)	< 0.001	1.77 (1.02–3.08)	0.044
Diameter (cm)				
≤ 2	1.00		1.00	
2–5	1.96 (1.47–2.62)	< 0.001	1.15 (0.85–1.55)	0.364
> 5	4.30 (2.99–6.17)	< 0.001	1.92 (1.26–2.95)	0.003
Differentiation				
Well-moderate	1.00		1.00	
Poor	1.32 (0.98–1.77)	0.064	1.03 (0.77–1.39)	0.833
Lymphovascular invasion				
No	1.00			
Yes	2.94 (2.27–3.81)	< 0.001		
ypT				
T0	1.00			
T1	1.68 (0.57–4.93)	0.342		
T2	2.11 (0.79–5.64)	0.138		
T3	3.94 (1.56–9.94)	0.004		
T4	7.04 (2.89–17.16)	< 0.001		
ypN				
N0	1.00			
N1	1.95 (1.30–2.93)	0.001		
N2	3.01 (2.02–4.47)	< 0.001		
N3	7.46 (5.28–10.53)	< 0.001		
ypTNM stage				
0	1.00	1.00	1.00	
I	0.94 (0.33–2.63)	0.901	0.92 (0.33–2.61)	0.880
II	2.36 (0.94–5.95)	0.067	1.98 (0.782–5.02)	0.150
III	7.76 (3.19–18.91)	< 0.001	5.32 (2.15–13.20)	< 0.001
Resection type				
Subtotal	1.00			
Total	1.76 (1.36–2.28)	< 0.001		
Adjuvant chemotherapy				
Yes	1.00		1.00	
No	1.58 (1.16–2.15)	0.003	1.42 (1.03–1.96)	0.034
Cycle of NACT				
> 3	1.00			
≤ 3	1.39 (0.85–2.28)	0.192		
Clavien–Dindo				
Grade 0–II	1.00		1.00	
Grade III–IV	1.36 (0.97–1.91)	0.073	1.03 (0.71–1.47)	0.889
CEA				
≤ 5.72	1.00			
> 5.72	2.30 (1.70–3.11)	< 0.001		
CA199				
≤ 15.00	1.00			
> 15.00	1.71 (1.32–2.21)	< 0.001		
CA724				
≤ 2.60	1.00			
> 2.60	1.81 (1.38–2.38)	< 0.001		
Post-NACT CTM				
0	1.00		1.00	
1	1.54 (1.06–2.23)	0.022	1.37 (0.94–2.00)	0.099
2	2.77 (1.87–4.10)	< 0.001	2.07 (1.37–3.10)	< 0.001
3	4.54 (2.85–7.22)	< 0.001	3.11 (1.91–5.07)	< 0.001

Values in parentheses are *95 percent confidence intervals. ASA, American Society of Anesthesiologists; BMI, Body Mass Index; CA19-9, carbohydrate antigen 19-9; CA72-4, carbohydrate antigen 72-4; CEA, carcinoembryonic antigen; CTM, combination of tumor markers; ASA, American Society of Anesthesiologists; ECOG, Eastern Cooperative Oncology Group; PFS, progression-free survival; NACT, neoadjuvant chemotherapy; CEA, carcinoembryonic antigen; CA19-9, carbohydrate antigen 19-9; CA72-4, carbohydrate antigen 72-4;

### Nomogram for prediction of PFS

**Fig. 4:**
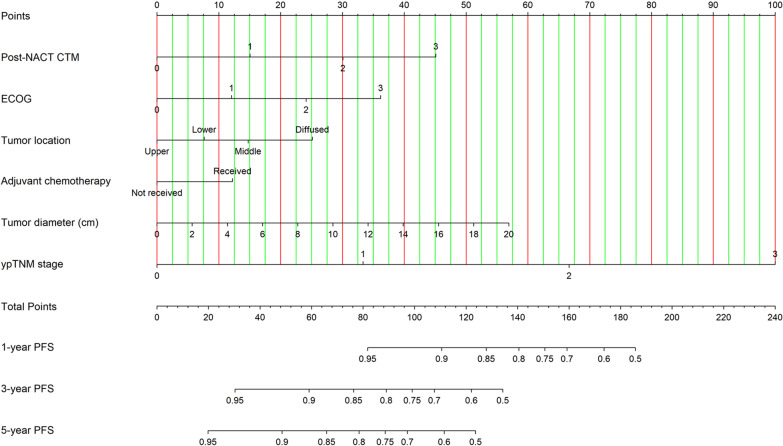
Post-neoadjuvant chemotherapy treatment nomogram for predicting 1-, 3- and 5-year progression-free survival. ypTNM stage = 0 stands for complete pathological response; NACT, neoadjuvant chemotherapy; PFS, progression-free survival

**Fig. 5:**
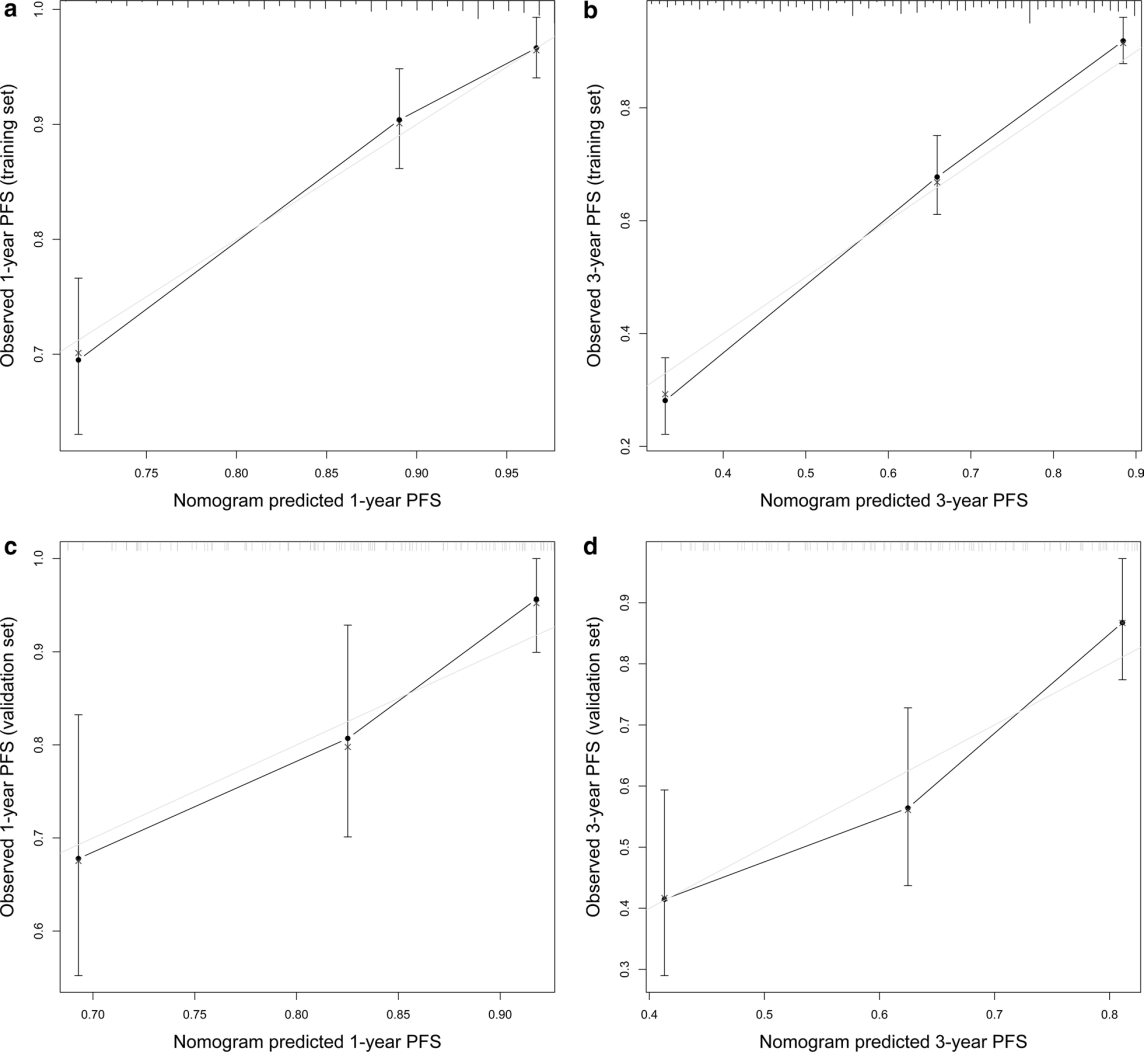
Calibration plots of the nomogram for 1-year in the training cohort (**a**), 3-year in the training cohort (**b**), 1-year in the validation cohort (**a**), 3-year in the validation cohort (**d**), progression-free survival. The x-axis represents nomogram-predicted survival, and the y-axis represents actual survival and 95% confidence intervals. Calibration curves analysis using 1000 bootstraps replicates predict survival in subcohorts of 1000 bootstrap samples. The dotted line represents the ideal correlation between predicted and actual PFS

To better predict the prognosis of clinical outcomes, a nomogram was established by involving all the independent prognostic factors above (Fig. 4). A higher total score reveals a higher probability of cancer progression. The nomogram showed that the ypTNM stage was the most significant predictor for PFS risk, followed by tumor diameter and post-NACT CTM. The C-index value of 0.762 (95% CI: 0.742–0.782) calculated for the nomogram model indicated a high prognostic prediction accuracy, outweighing the single value of ypTNM stage (C-index 0.706, 95% CI: 0.691–0.721) or post-NACT CTM (C-index 0.639, 95% CI: 0.620–0.658) based on proportional hazards model. The calibration plots for the probability of 1- and 3-year PFS also presented an optimal agreement between actual observation and the nomogram's prediction (Fig. 5a, b). The C-index in the validation group was 0.693 (95%CI, 0.630–0.756). The favorable calibration for 1-year and 3-year PFS were confirmed in the validation cohort (Fig. 5c, d).

### Performance of the post-NACT CTM for predicting pCR

pCR was confirmed in 34 cases (6.38%). The relationships between post-NACT CTM levels and clinicopathologic characteristics were summarized in Table 4. The higher level of post-NACT CTM scores was found significantly correlated with the positive lymph nodes number (*P* < 0.001), presence of ypN0 (*P* < 0.001) and pCR (*P* = 0.039). Univariate and multivariate analysis were performed by logistic regression to evaluate the predictive role of Post-NACT CTM, triplet therapy and NACT duration for pCR. The results showed that both duration of NACT (cycles ≥ 4, OR: 3.09, 95% CI: 1.30–7.33, *P* = 0.011) and lower post-NACT CTM score (2/3 vs. 1, OR: 2.75, 95% CI: 0.89–8.50, *P* = 0.078; 2/3 vs. 0, OR: 4.19, 95% CI: 1.33–13.14, *P* = 0.014) were strong predictors for pCR (Table 5). Simultaneously, we applied another multivariate model based on single tumor marker performance and found that CA72-4 revealed a significant association with pCR according to the cutoff value, while CEA and CA19-9 did not show any statistical difference in univariate regression (Table 5). The AUC values of post-NACT CTM score and CA72-4 were 0.628 (95% CI: 0.544–0.712, *P* = 0.003) and 0.601 (95% CI: 0.517–0.685, *P* = 0.019), respectively. The Spearman correlations were significant between post-NACT CTM and most predictor variables, but the correlation coefficients were low for all pairs (r < 0.25), which fortified the independence of prognostic ability of post-NACT CTM scores (Additional file 1: Table S2).

**Table 4:** Perioperative parameters and response information stratified by post-NACT CTM score after NACT in 533 patients

	All patients	CTM 0 (N = 140)	CTM 1 (N = 233)	CTM 2 (N = 116)	CTM 3 (N = 44)	*P*
Hospital stay (days)*	10 (9, 13)	10 (8, 13)	10 (9, 13)	11 (9, 14)	10 (9.5, 13.5)	0.137
Operative time (min)*	201 (174, 241)	199 (170, 233.5)	200 (172, 240)	203.5 (178, 245.5)	226 (179.5, 271)	0.072
Blood loss (ml)*	100 (100, 200)	100 (99.5, 150)	100 (100, 200)	100 (100, 200)	100 (100, 200)	0.064
The number of resected lymph nodes*	31 (23,40.5)	30.5 (22, 40)	30 (23, 40)	31 (24, 39.5)	31 (28, 40.5)	0.645
The number of positive lymph nodes*	1 (0, 5)	0 (0, 3)	1 (0, 4)	2 (0, 8)	4 (0, 11)	< 0.001
Clavien–Dindo						0.104
0–II	455 (85.37)	120 (85.71)	206 (88.41)	96 (82.76)	33 (75.00)	
III-IV	78 (14.63)	20 (14.29)	27 (11.59)	20 (17.24)	11 (25.00)	
LVI	177 (33.21)	36 (25.71)	68 (29.18)	56 (48.28)	17 (38.64)	0.001
ypT0	39 (7.32)	15 (10.71)	19 (8.15)	3 (2.59)	2 (4.55)	0.062^†^
ypN0	233 (43.71)	75 (53.57)	110 (47.21)	35 (30.17)	13 (29.55)	< 0.001
pCR	34 (6.38)	14 (10.00)	16 (6.87)	2 (1.72)	2 (4.55)	0.039^†^

Categorical variables are summarized with counts, percentage and *P* values based on chi-square tests; Continuous variables are summarized with median, IQR, and *P* values for Kruskal–Wallis test; CTM, combination of tumor markers; LVI, lymph vascular invasion; pCR, pathological complete response

^†^*P* value for Fisher’s exact test

**Table 5:** Univariate logistic regression analysis examining the influence of patient factors on pathologic complete response (pCR)

Values	Univariate analysis OR (95% CI)	*P*	Multivariate analysis OR (95% CI) (Model 1*)	*P*	Multivariate analysis OR (95% CI) (Model 2^†^)	*P*
Age > 60	1.28 (0.64–2.58)	0.481				
Male	1.74 (0.66–4.59)	0.265				
BMI ≤ 23.9	0.93 (0.46–1.89)	0.845				
Poor differentiation	1.75 (0.75–4.11)	0.196				
Linitis plastica	NA	NA				
NACT cycles ≥ 4	3.54 (1.56–8.05)	0.002	3.09 (1.30–7.33)	0.011	3.20 (1.36–7.55)	0.008
Triplet drug	4.73 (1.24–18.08)	0.023	3.09 (0.72–13.31)	0.130	3.31 (0.78–13.98)	0.103
CEA ≤ 5.72	2.06 (0.32–6.90)	0.241				
CA19-9 ≤ 15.00	1.53 (0.73–3.21)	0.259				
CA72-4 ≤ 2.60	2.29 (1.11–4.72)	0.025			2.32 (1.11–4.84)	0.025
Post-NACT CTM						
0	4.33 (1.39–13.49)	0.01	4.19 (1.33–13.14)	0.014		
1	2.88 (0.94–8.77)	0.060	2.75 (0.89–8.50)	0.078		
2/3	1.00		1.00			

*Model 1: adjusted for NACT cycles, triplet regimen and post-NACT CTM score; ^†^Model 2: adjusted for NACT cycles, triplet regimen CEA, CA19-9 and CA72-4; BMI, body mass index; CEA, carcinoembryonic antigen; CA19-9, carbohydrate antigen 19-9; CA72-4, carbohydrate antigen 72-4; CEA, carcinoembryonic antigen; CI, confidence interval; CTM, combination of tumor markers; OR, odds ratio; CI, confidence interval

### Changes of tumor markers and their correlation with PFS and pCR

**Fig. 6:**
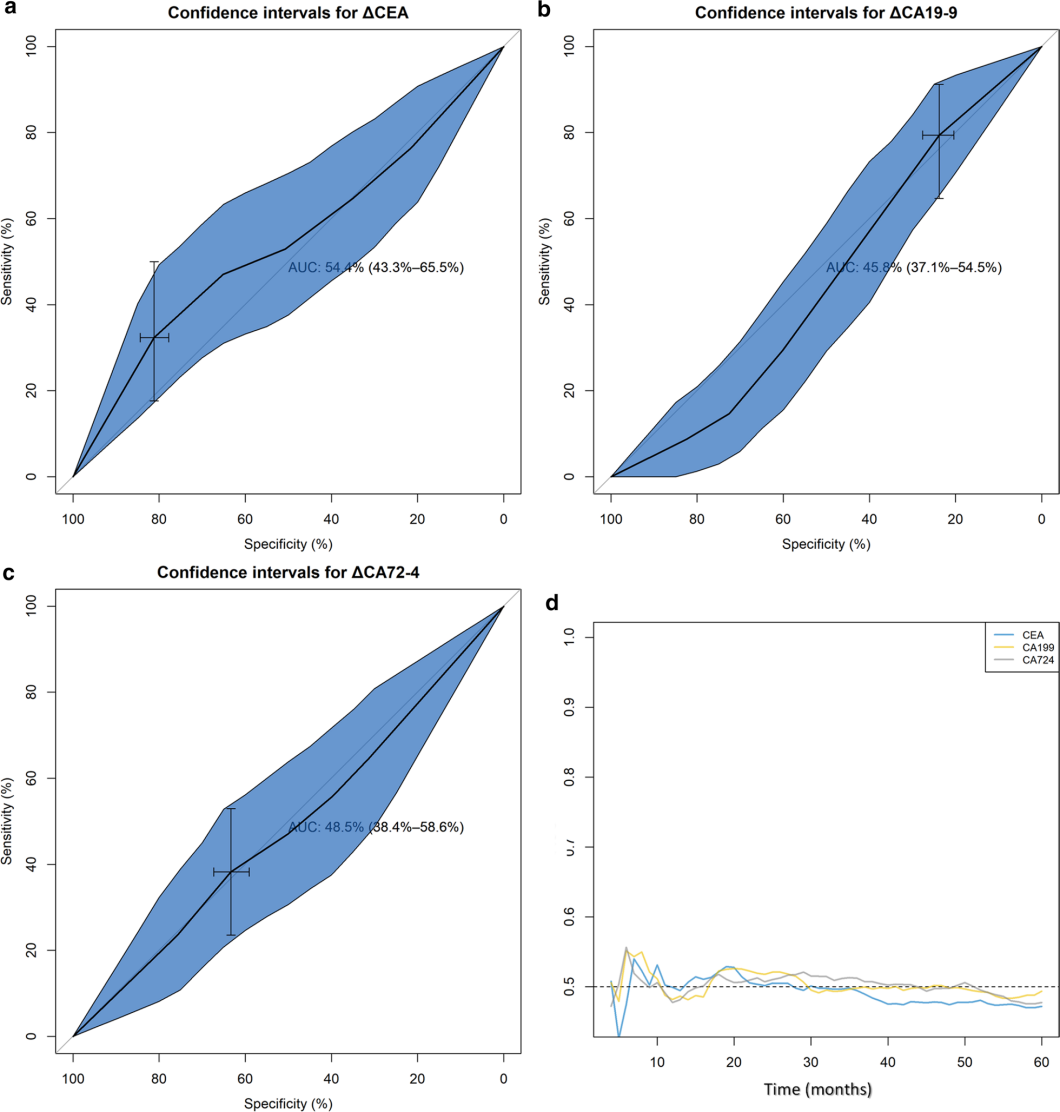
**a**–**c** ROC curves for the decrease of the three TMs to predict the pathological complete response (pCR) after neoadjuvant chemotherapy. The changes of TMs were compared with baseline level and are based on the following classification rules in CEA (**a**), CA19-9 (**b**), and CA72-4 (**c**) elevation > 50%, elevation > 20% but < 50%, elevation < 20%, decline < 20%, decline > 20% but < 50% and decline > 50% of the baseline levels. The figure shows the of these markers decrease of CEA (AUC: 0.544, 95% CI: 0.433–0.655), CA19-9 (AUC: 0.458, 95% CI: 0.371–0.545) and CA72-4 (AUC: 0.485, 95% CI: 0.384–0.586) was not statistically significant **c**. **d** The time-dependent AUC for prediction of risk of PFS for the level change in CEA, CA19-9, CA72-7 are also plotted. All three markers fluctuated around the reference line throughout the follow-up months, indicating there is no prognostic value for PFS among changes of CEA, CA19-9, and CA72-4 during NACT period

Median time from the initiation of NACT to the operation was 97 days (range: 78–119). However, no differences were found between the baseline and post-NACT concentrations of CEA, CA19-9 and CA72-4 either for the whole sample or stratified by ypTNM stage (Tables 6, 7 and 8). No efficient AUC of CEA, CA19-9 or CA72-4 was observed in pCR rate (Fig. 6a–c) or in the prediction of progression according to a ranking-based evaluation using ROC-AUC and time-dependent ROC-AUC (Fig. 6d).

**Table 6:** Treatment progress and changes in the serum carcinoembryonic antigen (CEA) level among different stages

Stage	Baseline CEA levels	Post CEA levels	P*	Decreased	*P*^*†*^	Decreased > 20%	*P*^*†*^
All patients	2.45 (1.46, 5.20)	2.42 (1.63, 4.21)	0.651	264 (49.53)	0.335	190 (35.65)	0.027
0	2.86 (1.3, 13.67)	2.41 (1.61, 4.05)	0.303	18 (52.94)		16 (47.06)	
I	2.23 (1.55, 3.98)	2.33 (1.64, 3.97)	0.973	43 (41.75)		26 (25.24)	
II	2.37 (1.38, 5.07)	2.30 (1.60, 3.98)	0.793	79 (49.69)		53 (33.33)	
III	2.58 (1.48, 7.1)	2.52 (1.65, 4.68)	0.923	124 (52.32)		95 (40.08)	

*P value for Wilcoxon test; ^†^*P* value for chi-square test

**Table 7:** Treatment progress and changes in the serum carbohydrate antigen 19-9 (CA19-9) level among different stages

Stage	Baseline CA19-9 levels	Post CA19-9 levels	P*	Decreased	*P*^*†*^	Decreased > 20%	*P*^*†*^
All patients	11.23 (6.31, 24.55)	12.88 (7.24, 24.64)	0.144	209 (39.21)	0.186	142 (26.64)	0.072
0	8.87 (5.71, 16.31)	12.02 (7.51, 18.59)	0.303	10 (29.41)		5 (14.71)	
I	9.75 (6.27, 16.88)	12.58 (7.74, 17.75)	0.177	33 (32.04)		20 (19.42)	
II	11.78 (6.56, 23.12)	12.32 (7.03, 25.09)	0.619	67 (42.14)		48 (30.19)	
III	12.15 (6.60, 33.49)	13.43 (7.17, 27.80)	0.533	99 (41.77)		69 (29.11)	

*P value for Wilcoxon test; ^†^*P* value for chi-square test

**Table 8:** Treatment progress and changes in the serum carbohydrate antigen 72-4 (CA72-4) level among different stages

Stage	Baseline CA72-4 levels	Post CA72-4 levels	P*	Decreased	*P*^*†*^	Decreased > 20%	*P*^*†*^
All patients	2.99 (1.45, 8.64)	2.92 (1.54, 7.12)	0.801	272 (51.03)	0.594	209 (39.21)	0.592
0	2.12 (1.06, 5.07)	2.03 (1.29, 3.71)	0.951	16 (47.06)		14 (41.18)	
I	2.03 (1.26, 4.56)	2.15 (1.33, 5.30)	0.420	48 (46.60)		37 (35.92)	
II	2.84 (1.31, 7.62)	2.92 (1.50, 6.91)	0.566	80 (50.31)		58 (36.48)	
III	3.67 (1.96, 12.12)	3.69 (1.64, 8.17)	0.564	128 (54.01)		100 (42.19)	

*P value for Wilcoxon test; ^†^*P* value for chi-square test

## Discussion

Unlike AFP for hepatic cell carcinoma or CA125 for ovarian tumors, the relationship between TMs and the prognosis of the GC was unclear. This is largely because there is no conclusive evidence that TMs expression levels can exactly reflect tumor burden. Of the three TMs in the present study, CEA is an oncofetal glycoprotein of the cell surface involved in intracellular adhesion during the fetal development of gastrointestinal tissue [20]. CEA's overexpression can be particularly observed in patients with various malignancies, including adenocarcinomas of the gastrointestinal tract (stomach, colon, rectum, and pancreas). It is commonly hypothesized that the overexpressed CEA protein occupies cell membranes' surface and prevents normal growth inhibition and cellular differentiation, which finally leads to tumor progression [21, 22]. CA19-9, on the other hand, is a sialylated Lewis blood group antigen but is widely expressed in gastrointestinal malignancies. The biological functions of CA19-9 are still poorly defined. As a mucin antigen with high molecular weight, CA19-9 is hypothesized to act as an anti-adhesive molecule contributing to tumor migrations and to locally inhibit T cell-mediated antitumor response [23, 24]. Moreover, the prominent predictive ability of CA19-9 for pancreatic cancer indicated the possibility to measure tumor biology from a quantitative view [11, 25]. CA72-4 was initially designated as a tumor-associated glycoprotein-72 which has been found in various epithelial malignancies tumors as well as benign gastritis [26, 27]. Although with obscure biological functions, CA72-4 appears to be more accurate than CEA and CA19-9 in detecting lymph node status and progression in GC [28]. Moreover, the serum level of CA72-4 correlating with the stage of tumor has been confirmed by previous studies [26, 29, 30]. Taken together, all these TMs are nonspecific for GC, but the elevation of each TM should mechanistically accompany the tumor growth, infiltration, and spreading into the blood circulation. With the cumulative clinical evidence, it is justifiable to suppose that CEA, CA19-9 and CA72-4 could be used conjunctively in reflecting tumor burden for LAGC and predicting prognosis [31, 32].

For TMs being as prognostic factors in LAGC patients undergoing NACT, there are two distinct questions to be addressed first. One problem is that treatment duration, dosage and chemosensitivity are not equalized in each patient. To eradicate confounders surrounding NACT settings, the post-NACT TMs values should be more logical with explicit clinical meaning used as benchmark value of TMs in predicting patients prognosis and tumor response rather than pretreatment values. The other question is the indication of changed TMs values during NACT. As current NACT protocols are far from satisfying in treating LAGC, the decreased values of TMs are not always magnificent and may not be easily interpreted [8, 9]. On the other hand, the alteration of TMs is considered to reflect the chemotherapy efficacy and chemosensitivity of the tumors, but not the residual tumor burden, although it can be employed to indicate prognosis to some degree [33, 34]. In the light of these, a post-NACT, comprehensive, cross-sectional benchmark value for TMs was introduced in the current study.

Because the low sensitivities and specificities, using "normal range" as cutoff values, restrict their clinical application, the predictions based on the serum TMs and combined method were investigated using time-dependent ROC curves for cumulative PFS. Relating to CA19-9 and CA72-4, the cutoff values were within the "normal range". This is reasonable as current ranges of TMs are mainly designed for screening the general population instead of predicting tumor progression in already diagnosed patients [35]. Fluctuations in TMs expression frequently occur in patients following NACT, which is also likely to require a reframing of TMs norms and standards. Ma et al. investigated a cohort of 154 LAGC patients with tumor regression grade 0–1 and found that CEA lower than 5.0 ng/ml after NACT greatly improved patients prognosis [36]. In other words, the criteria of each TM may be varied in terms of different usage.

We did not evaluate the efficacy of TMs on overall survival as some previous work did [32, 37]. Considering that patients' long-term survival relied on the treatment after recurrence, the results of post-NACT TMs on OS should be affected by miscellaneous factors and be over-interpreted. We also used dynamic AUC to illustrate the consistent advantages in post-NACT CTM for predicting the risk of progression. This avoids data-driven analysis and enhances transparency. Furthermore, the results of the log-rank test and Cox regression indicated that post-NACT CTM could classify patients into four independent groups with good discrimination of PFS.

Although nomographs were widely publicized, less work has been done on NACT patients following the 8th AJCC ypTNM system. The nomogram profiling for NACT population was firstly introduced in our previous study [38]. However, the information of TMs in our previous work was not available. Following that ypTNM stage, tumor location, and BMI were independent prognostic factors, the present work complemented our previous with a satisfactory C-index of 0.762 based on internal validation and good continuity in the validation cohort. Of note, the post-NACT CTM = 3 contributed the third-highest HR to the model. This indicates that the combined diagnosis of tumor marker serves as a potentially strong prognostic indicator for disease progression after NACT. Interestingly, as we conducted the correlation analysis, all the included covariates revealed either weak or non-significant associations with post-NACT CTM scores. The reason for its independent prognostic value might be credited to TMs' unique diagnostic advantages. In measuring residual tumor burden, not only TMs can reflect viable tumor mass, but it may indicate the amount of circulating tumor cells which can further forecast metastases [39, 40].

It has been confirmed that there is a strong association between pCR and long-term survival or recurrence [41, 42]. The pCR rate of gastric cancer patients after NACT is likely to be affected by the tumor location, differentiation, the Lauren classification and type of chemotherapy regimens and cycles [43]. In the realm of tumor markers, results are not conclusive. Either the baseline or the changes of TMs might be a potential indicator for complete pathological response. Sun et al. retrospectively reviewed 184 GC patients with NACT and found pretreatment CEA and CA72-4 change were associated with a higher response rate [33]. Chen et al. first proposed a nomogram method giving a systemic evaluation based on pretreatment parameters in patients receiving NACT and found only CEA had prognostic value on pCR [44]. Interestingly, as opposed to our studies, higher CEA (> 5.0 ng/ml) is associated with higher probability of pCR in their study. The authors hypothesized that elevated CEA is associated with heavier tumor load and faster tumor growth rate, and indicate that tumors are more susceptible to chemotherapy. We believe their hypothesis should be testified by the post-NACT TMs level under the premise that cross-sectional levels of TMs reflect residual tumor load. Contrary to Chen et al., the lower post-NACT level of CA72-4 and post-NACT CTM score significantly correlated with pCR rate, while CEA, CA19-9 and TMs’ change did not reveal statistical relevance. Our results advocate that post-NACT CTM reveals residual tumor burden which can furtherly predict tumor pathological response. However, there is another equally important possibility that should not be neglected. As NACT for gastric cancer is of limited benefit, usually with pCR rate less than 10%, studies on pCR prediction are based on fairly low rate of positive events and insufficient sample size [45–47]. We believe this could be the main reason that some of previous results are with low repeatability. To achieve a convincing result or model, either the sample size should multiply or a combined diagnosis method should be introduced, which could cut down the number of covariates and therefore stabilize variances. In the current study, on the premise of the capacity, we adopted the latter one as a solution.

As dynamic monitoring of TMs change is considered as a regular practice in screening progression or recurrence. We also conducted a comparison between the pretreatment TMs and post-NACT TMs, aiming to find clues on prognosis. Unfortunately, contrary to Sun et al., no statistical differences were found between the baseline and post-NACT levels of three TMs. Nor did we find altering of these TMs could serve as a prognostic indicator for pathological responses or disease progression. We suggest that the gastric adenocarcinoma is generally less responsive, resulting in over one-third of poor-responders after NACT, relatively short treatment duration may not meet the window that could reflect the treatment efficacy (around half of our patients received two cycles of NACT). It should be noted that surgical resection is the only curative approach for GC, and sometimes there might be tumor markers surge after initiation of chemotherapy [5, 48]. While predicting pathological response during NACT in TMs' change remained ambiguous, the post-NACT timepoint level is of clinical significance.

We acknowledge that there were some limitations to our study. First, the study is restricted by its single-center retrospective nature. Second, although the definition of cutoff values followed a systemic design, a population-based study is required to set our cutoff values more accurately. Third, although a validation set was formed, there lacked large prospective studies for validation, and some inflammatory and nutritional markers like neutrophil-to-lymphocyte ratio or prognostic nutrition index were not evaluated in our regression model [49]. Last but not least, the change of TMs may not be fully investigated in our study. We are preparing to conduct long-term surveillance with controlling of confounders more strictly to address this question further.

## Conclusions

In conclusion, we have demonstrated that post-NACT CTM showed a favorable accuracy as an independent predictor of progression as well as pathological response in LAGC patients with neoadjuvant chemotherapy. As a simple but cost-effective method, we believe it points to a way of prognostic prediction and guiding treatment strategies in LAGC patients who received NACT. Limited by its retrospective nature and capacity, our issues serve as the triggers of yet prospective population-based studies to refine our criteria in the future.

## Supplementary Information

**Additional file 1**. **Table S1**. Dosage and schedule of the treatment regimen; **Table S2**. Spearman correlation analysis between the post-NACT CTM and potential prognostic factors

**Additional file 2**.** Fig. S1**. Time-dependent ROC curves for progression-free survival (PFS) at 1st, 3rd, and 5th year time points are plotted for (A) CEA, (B) CA19-9, (C) CA72-4, (D) post-NACT CTM. The cutoff values for CEA, CA19-9, CA72-4 are 5.72 (sensitivity 27.49%, specificity 90.33%), 15.00 (sensitivity 54.9%, specificity 66.91%) and 2.60 (sensitivity 67.11%, specificity 53.90%) which are based on 3-year PFS using Kaplan–Meier method.

## Data Availability

The datasets during and/or analyzed during the current study are available from the corresponding author on reasonable request.

## Abbreviations

AFP: Alpha fetoprotein
AJCC: American Joint Committee on Cancer
ASA: American Society of Anesthesiologists
AUC: Area under curve
BMI: Body mass index
CA125: Cancer antigen 125
CA19-9: Cancer antigen 19-9
CA72-4: Cancer antigen 72-4
CEA: Carcinoembryonic antigen
CTM: Combination of serum tumor markers
GC: Gastric cancer
IPCW: Inverse probability of censoring weighting
JGCA: The Japanese Gastric Cancer Association
LAGC: Locally advanced gastric cancer
LVI: Lymph-vascular invasion
NACT: Neoadjuvant chemotherapy
OS: Overall survival
pCR: Pathological complete response
PFS: Progression free survival
ROC: Receiver operating curve
TMs: Tumor markers
