# Supplementary material for: Combination of tumor markers predicts progression and pathological response in patients with locally advanced gastric cancer after neoadjuvant chemotherapy treatment
Source: BMC Gastroenterol. 2021 Jul 10;21:283. doi: 10.1186/s12876-021-01785-7 (PMC8272383; doi:10.1186/s12876-021-01785-7)
Supplement: Supplementary file 1 — Additional file 1. Table S1. Dosage and schedule of the treatment regimen; Table S2. Spearman correlation analysis between the post-NACT CTM and potential prognostic factors [file 12876_2021_1785_MOESM1_ESM.docx]

Table S1 Dosage and schedule of the treatment regimen

| Regimen | Drug dosage | Schedule | Duration |
| --- | --- | --- | --- |
| SOX | Oxaliplatin: 130mg/m2 IV | Days 1 | Q3wk, up to 8 cycles |
|  | S-1: 80mg (<1.25m2); 100mg (1.25-1.5m2); 120mg (>1.5m2) PO | Days 1-14 |  |
| CapeOX | Oxaliplatin: 130mg/m2 IV | Days 1 | Q3wk, up to 8 cycles |
|  | Capecitabine: 1000 mg/m2 PO | Days 1-14 |  |
| FOLFOX | Oxaliplatin: 85 mg/m2 IV | Days 1 | Q2wk, up to 12 cycles |
|  | Leucovorin: 400 mg/m2 IV | Days 1 |  |
|  | 5-Fu: 400 mg/m2 IVP | Days 1 |  |
|  | 5-Fu (continuous): 2400-3000 mg/m2 IV | Days 1-2 |  |
| CS | Cisplatin: 80 mg/m2 IV | Days 1 | Q3wk, up to 8 cycles |
|  | S-1: 80mg (<1.25m2); 100mg (1.25-1.5m2); 120mg (>1.5m2) PO | Days 1-14 |  |
| PS | Paclitaxel: 150 mg/m2 IV | Days 1, 8 | Q3wk, up to 8 cycles |
|  | S-1: 80mg (<1.25m2); 100mg (1.25-1.5m2); 120mg (>1.5m2) PO | Days 1-14 |  |
| PX | Paclitaxel: 150 mg/m2 IV | Days 1, 8 | Q3wk, up to 8 cycles |
|  | Capecitabine: 1000 mg/m2 PO | Days 1-14 |  |
| IRIS | Irinotecan: 180 mg/m2 IV | Days 1 | Q3wk, up to 8 cycles |
|  | S-1: 80mg (<1.25m2); 100mg (1.25-1.5m2); 120mg (>1.5m2) PO | Days 1-14 |  |
| EOX | Epirubicin 50 mg/m2 IV | Days 1 | Q3wk, up to 6 cycles |
|  | Oxaliplatin 130 mg/m2 IV | Days 1 |  |
|  | Capecitabine: 625 mg/m2 PO | Days 1-14 |  |
| DCF | Paclitaxel: 75 mg/m2 IV | Days 1 | Q3wk, up to 6 cycles |
|  | Cisplatin: 60mg/m2 IV | Days 1 |  |
|  | 5-Fu : 750 mg/m2 IV | Days 1-5 |  |
| POS | Paclitaxel: 120 mg/m2 IV | Days 1 | Q2wk, up to 6 cycles |
|  | Oxaliplatin: 185mg/m2 IV | Days 1 |  |
|  | S-1: 40mg (<1.25m2); 50mg (1.25-1.5m2); 60mg (>1.5m2) PO | Days 1-7 |  |

Abbreviations: PO, by oral; IV, intravenous

Table S2 Spearman correlation analysis between CTM and potential prognostic factors

| Variables | Post-NACT CTM socres | |
| --- | --- | --- |
|  | Spearman correlation, r | *P* value |
| BMI | 0.010 | 0.818 |
| ECOG | 0.122 | 0.005 |
| Location | -0.055 | 0.205 |
| Diameter | 0.203 | <0.001 |
| Differentiation | -0.053 | 0.219 |
| LVI | 0.154 | <0.001 |
| ypT | 0.219 | <0.001 |
| ypN | 0.214 | <0.001 |
| ypTNM stage | 0.230 | <0.001 |
| Cycles of NACT | 0.004 | 0.920 |
| Number of positive lymph nodes | 0.208 | <0.001 |
| Triplet drug | -0.013 | 0.766 |

Abbreviations: ASA, American Society of Anesthesiologists; BMI, Body Mass Index; CTM, combination of tumor markers; ECOG, Eastern Cooperative Oncology Group; LVI, lymphovascular invasion; NACT, neoadjuvant chemotherapy
