# Supplementary figures and images for: Combination of tumor markers predicts progression and pathological response in patients with locally advanced gastric cancer after neoadjuvant chemotherapy treatment
Source: BMC Gastroenterol. 2021 Jul 10;21:283. doi: 10.1186/s12876-021-01785-7 (PMC8272383; doi:10.1186/s12876-021-01785-7)

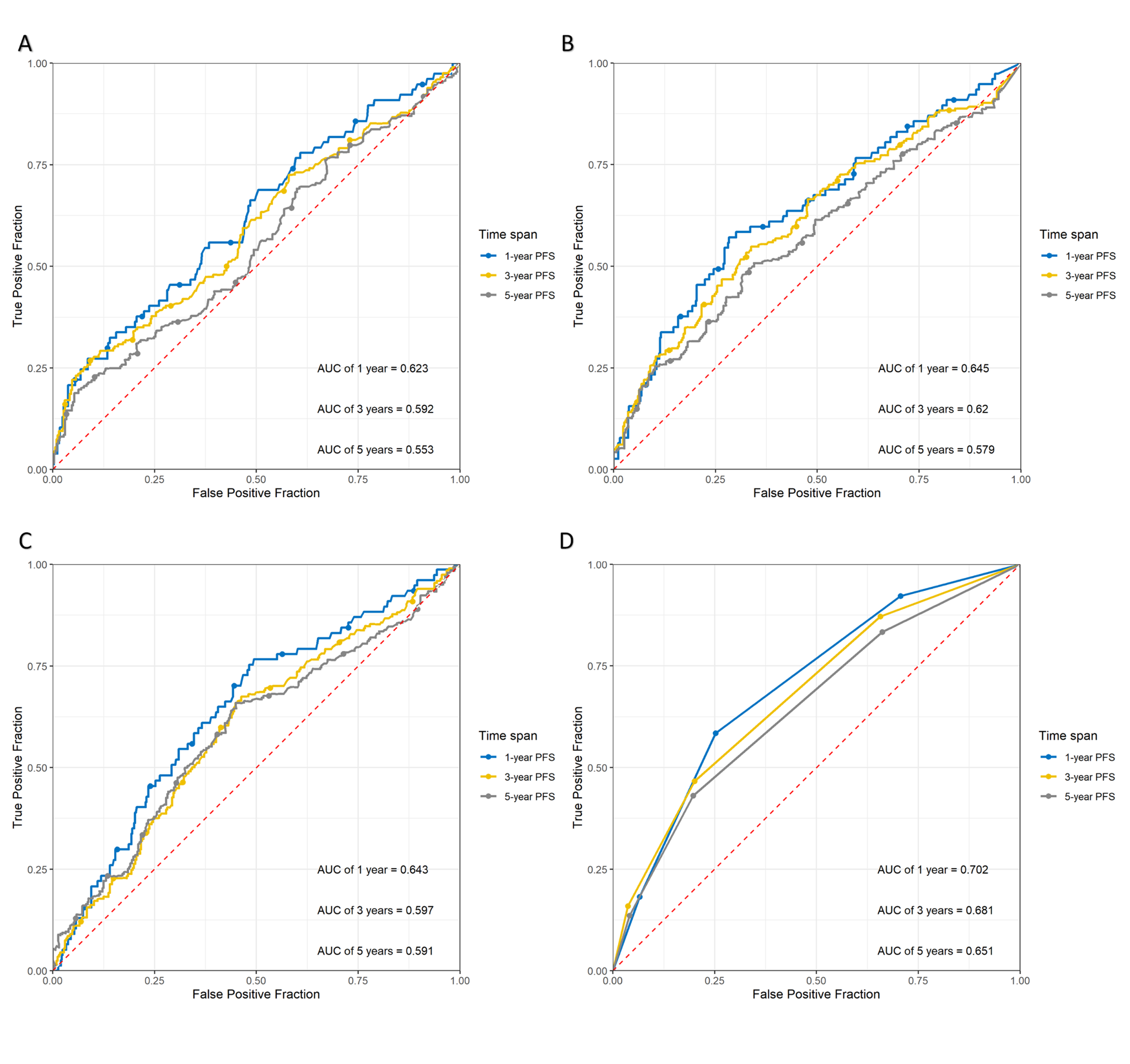

Supplement: Supplementary file 2 — Additional file 2. Fig. S1. Time-dependent ROC curves for progression-free survival (PFS) at 1st, 3rd, and 5th year time points are plotted for (A) CEA, (B) CA19-9, (C) CA72-4, (D) post-NACT CTM. The cutoff values for CEA, CA19-9, CA72-4 are 5.72 (sensitivity 27.49%, specificity 90.33%), 15.00 (sensitivity 54.9%, specificity 66.91%) and 2.60 (sensitivity 67.11%, specificity 53.90%) which are based on 3-year PFS using Kaplan–Meier method. [file 12876_2021_1785_MOESM2_ESM.tif]
